# LumenGSLAM: online physically based rendering with Gaussian Splatting for robust endoscopic reconstruction and tracking

**DOI:** 10.1007/s11548-026-03660-w

**Published:** 2026-05-13

**Authors:** Francesco Leni, Chiara Lena, Zhehua Mao, Sierra Bonilla, Luca Carlini, Danail Stoyanov, Elena De Momi, Sophia Bano

**Affiliations:** 1 Department of Electronics, Information, and Bioengineering, Politecnico di Milano, Via Giuseppe Ponzio, 34, Milan, 20133 Italy; 2 UCL Hawkes Institute and Department of Computer Science, University College London, London, UK

**Keywords:** Gaussian splatting, Endoscopy, SLAM, 3D reconstruction, Physically based rendering

## Abstract

**Purpose:**

Realistic online 3D reconstruction from endoscopic video is essential for intraoperative inspection and navigation. However, existing approaches often neglect realistic light modeling, rely on offline optimization, or depend on fragile photometric tracking, thereby limiting physically plausible rendering and stable tracking. This study addresses these limitations through an online framework enabling stable tracking and photorealistic endoscopic rendering.

**Methods:**

We propose LumenGSLAM, an online RGB-D Gaussian Splatting framework for highly texturized dense reconstruction of endoscopic scenes. The method leverages dense depth input to enable stable geometry estimation and photorealistic appearance modeling through per-Gaussian physically based rendering (PBR). Surface-aligned Gaussian initialization and per-parameter gradient scaling are introduced to enhance anatomical fidelity and geometric consistency. Robust camera pose estimation is achieved via a Gaussian-coupled feature-based tracking module using SuperPoint/LightGlue and Perspective-n-Point (PnP), ensuring reliable localization under rapid motion and challenging illumination conditions.

**Results:**

Evaluated on C3VD and SCARED datasets, LumenGSLAM achieves superior online reconstruction and tracking performance. It attains PSNR = 30.6, SSIM = 0.89, and LPIPS = 0.23 on C3VD, outperforming all online baselines and approaching state-of-the-art offline PR-ENDO quality. In tracking, it delivers the lowest Absolute Trajectory Error (ATE = 0.93 mm) and Rotational Error (ARE = 0.98$^{\circ }$), demonstrating robustness even under large inter-frame motions.

**Conclusion:**

LumenGSLAM establishes a new benchmark for online RGB-D endoscopic reconstruction, achieving photometrically consistent and anatomically accurate mapping through explicit light modeling and geometry-aware Gaussian optimization. Its robustness makes it a promising candidate for intraoperative navigation and future extensions toward dynamic tissue modeling. Project page: https://github.com/FrancescoLeni/LumenGSLAM.

**Supplementary Information:**

The online version contains supplementary material available at 10.1007/s11548-026-03660-w.

## Introduction

Endoscopy plays a central role in minimally invasive surgery (MIS), enabling clinicians to inspect internal organs through natural or surgically created access routes. While this approach reduces patient trauma and accelerates recovery, the confined operative space imposes strict constraints on the imaging device, which is typically monocular and relies on fisheye optics to maximize the field of view. This setup, however, introduces strong optical distortions and eliminates true depth perception, posing major challenges for navigation and spatial understanding during procedures. Having a system capable of producing a high-quality reconstruction of the anatomy simultaneously with the ongoing procedure would be extremely beneficial, as it would provide the operator with a global overview of the explored cavity, enhancing perception and enabling virtual navigation. Thus, considerable research efforts is focusing in this direction [1].

To achieve consistent environment reconstruction and camera tracking, Simultaneous Localization and Mapping (SLAM) has demonstrated excellent performance in surgical environments. However, since the primary objective of these systems is accurate localization, the environment is typically represented by a sparse set of points, just sufficient to support tracking. As a result, additional post-processing steps are required to obtain a coherent 3D reconstruction [2, 3]. When the main objective is high-quality, texturized reconstruction of the environment, recent advancements in Neural Rendering [4] have emerged as a promising solution. Particularly, Neural Radiance Fields (NeRF) [5] enable dense volumetric reconstructions by learning an implicit scene representation through neural networks. Those methods deliver high-quality reconstructions, but they suffer from long training and slow inference, limiting their feasibility in real clinical contexts [6, 7].

More recently, 3D Gaussian Splatting (3DGS) [8] has emerged as a promising alternative for scene representation, enabling rapid deployment while maintaining competitive reconstruction and texture quality. Many recent approaches for endoscopic reconstruction and tracking assume access to dense depth information, typically obtained from specialized sensing setups or auxiliary depth estimation modules, to support geometric estimation. Gaussian Pancakes [9] introduces a surface loss term to regularize splats, while PR-ENDO [10] explicitly models illumination through an MLP integrated into a Physically Based Rendering (PBR) formulation. Despite the high visual fidelity, those pipelines remain offline, as they require an initial reconstruction and camera trajectory obtained from external SLAM frameworks. To move toward online operation, EndoGSLAM [11] pioneered the use of Gaussian Splatting as a geometric representation for SLAM in colonoscopy. This model produces good reconstruction, but texture quality is poor due to reliance on simplified isotropic Gaussians, and tracking is limited by pure photometric optimization, which may hinder robustness under rapid motion or challenging illumination conditions.

Building on these advances, we propose **LumenGSLAM**, an online dense RGB-D reconstruction framework that leverages Gaussian Splatting as its core scene representation for highly texturized 3D modeling and photorealistic rendering in endoscopic scenes. The method leverages dense depth input to support stable geometry estimation. Unlike offline reconstruction pipelines [8–10], LumenGSLAM operates fully online, incrementally expanding and refining a surface-aligned Gaussian map while jointly estimating camera pose at each frame.

To improve visual fidelity and physical consistency, light and tissue appearance are explicitly decoupled through per-Gaussian PBR modeling, avoiding the overhead of MLP-based lighting modules as in [10]. To ensure stable online reconstruction under rapid motion and complex illumination, we introduce a Gaussian-coupled keypoint-based tracking strategy that enhances pose robustness compared to photometric-only optimization.

The main contributions of LumenGSLAM can be summarized as follows:

- An online RGB-D framework for highly texturized dense 3D reconstruction and photorealistic rendering via Gaussian Splatting with per-Gaussian PBR modeling.
- Surface-aligned Gaussian initialization with per-parameter gradient scaling to enhance anatomical fidelity and geometric consistency during online optimization.
- A Gaussian-coupled keypoint tracking module designed to provide robust and accurate pose estimation as an enabling component for stable reconstruction under wide motion and challenging lighting conditions.

**Fig. 1:**
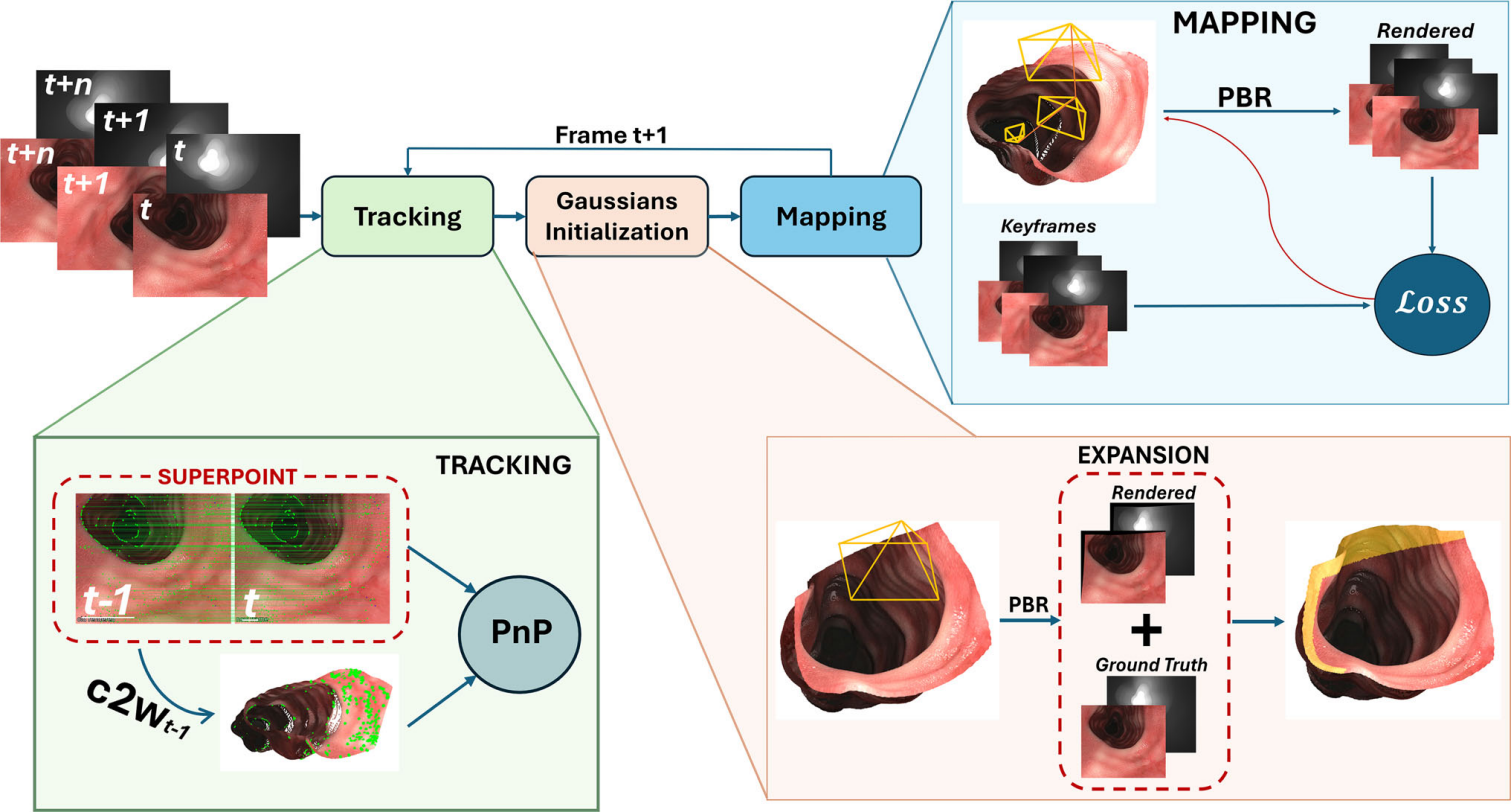
Overview of LumenGSLAM pipeline: Given streaming RGB-D input frames, the model estimates the pose of the current frame *t* through 3D-2D point registration. Then the Gaussian population is expanded in unseen areas and, finally, the global map is optimized over a selected set of keyframes

## Methods

### Overview

LumenGSLAM is an online RGB-D visual SLAM pipeline that adopts Gaussian Splatting [8] as its core scene representation, achieving 100+ fps rendering speed (more details regarding runtime performance are reported in App. B of Suppl.). The system, illustrated in Fig. 1, is organized into three main modules: taking as input an RGB-D stream, a tracking block first estimates the current camera pose using a Gaussian-coupled keypoint-based approach; an expansion block then integrates information from the same current frame to incrementally enlarge the global map by adding new surface-aligned Gaussians. Finally, a mapping block performs online optimization of global map parameters over a selected set of keyframes. To further enhance rendering quality, LumenGSLAM incorporates explicit light modeling through per-Gaussian PBR and a per-parameter gradient scaling.

### Surface-aligned Gaussians initialization

In LumenGSLAM, the scene is represented as a collection of learnable 3D Gaussians [8], each parameterized by its center μ, covariance (expressed through a rotation quaternion and a scaling vector), opacity α, and base color (albedo) encoded via spherical harmonic (SH) coefficients. Inspired by [10], we further augment each Gaussian with reflectance $\textit{F}_0$ and roughness *r*, experimentally initialized to 0.035 and 0.3 respectively, and enabling per-Gaussian modeling of optical properties and thereby supporting realistic light behavior through PBR.

Different from previous works, which use surface normals to regularize the loss [9, 10], it has been found more effective for PBR convergence to treat surface normals as geometric priors. For each frame, these normals are computed online from the spatial gradients of the input depth map. Then, during initialization, the local *z*-axis of each new Gaussian’s covariance is explicitly aligned with the corresponding surface normal. New surface-aligned Gaussians are introduced whenever a new frame becomes available and back-projected from the available depth map. For the first frame, all pixels are considered to initialize the map. For subsequent frames, only pixels from previously unseen regions are added, specifically, those with alpha-blended opacity below 0.9 or with ground-truth depth closer than the current estimate, indicating newly observed structures.

**Fig. 2:**
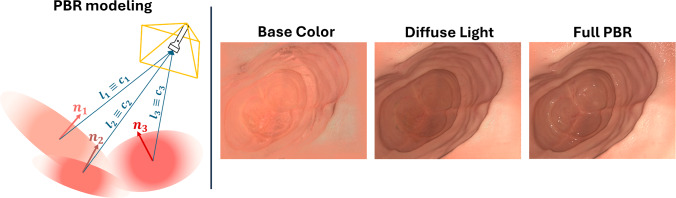
Graphical interpretation of PBR and examples of light decoupling

### Physically based rendering

As pictured in Fig. 2, color and illumination are effectively decoupled. Before rendering a new image, the color of each Gaussian is computed based on the camera view and the light source, modeled and optimized as a scene parameter. The light contribution at each Gaussian is evaluated as a combination of diffuse $f_{\text {diff},i}$ and specular components $f_{\text {spec},i}$. Unlike PR-ENDO [10], which estimates diffuse light via an MLP, we purely rely on Lambertian illumination. The final color is obtained by computing the Bidirectional Reflectance Distribution Function (BRDF) [12] function for each of the *i*-th visible Gaussian, as formulated in equation (1):

1 $$\begin{aligned} f_{\text {diff},i}&= a_i \, (\textbf{n}_i \cdot \textbf{l}_i), \nonumber \\ f_{\text {spec},i}&= \frac{D_i \, G_i \, F_i}{4 \, (\textbf{l}_i \cdot \textbf{n}_i) (\textbf{c}_i \cdot \textbf{n}_i)}, \nonumber \\ \textit{BRDF}_{i}&= (1-F_i)f_{\text {diff},i} + f_{\text {spec},i}, \end{aligned}$$

where $\textbf{n}_i$ denotes the normal of the *i*-th Gaussian, $\textbf{l}_i$ and $\textbf{c}_i$ are the light and camera directions (coincident in our implementation), $a_i$ is the diffuse albedo. $D_i$ is the microfacet distribution, modeled through Trowbridge–Reitz GGX, $G_i$ and $F_i$ are respectively geometric attenuation and Fresnel reflection, modeled via Schlick–Beckmann approximation. These are expressed as follows, where $\textbf{H}$ denotes the half-vector between light and camera directions:

2 $$\begin{aligned} F_i= &   F_{0,i} + (1 - F_{0,i}) (1 - (\textbf{l}_i \cdot \textbf{H}_i))^5, \end{aligned}$$

3 $$\begin{aligned} D_i= &   \frac{r_i^4}{\pi \left[ (\textbf{n}_i \cdot \textbf{H}_i)^2 (r_i^4 - 1) + 1 \right] ^2}, \end{aligned}$$

4 $$\begin{aligned} G_i= &   \frac{\textbf{n}_i \cdot \textbf{l}_i}{(\textbf{n}_i \cdot \textbf{l}_i)(1 - k) + k} \cdot \frac{\textbf{n}_i \cdot \textbf{c}_i}{(\textbf{n}_i \cdot \textbf{c}_i)(1 - k) + k},\nonumber \\  &   \quad \text {with } k = \tfrac{r_i^2}{2}. \end{aligned}$$

### Gaussians-coupled tracking

Unlike previous models [11, 13, 14], LumenGSLAM adopts a feature-based tracking strategy, avoiding unstable purely photometric pose optimization, which may easily get stuck in local minima in case of locally homogeneous geometry or highly dynamic illumination [15]. State-of-the-art SuperPoint [16] is employed to provide repeatable keypoints and robust local descriptors, together with LightGlue [17], a transformer-based matcher that selects reliable correspondences across views. To ensure consistent tracking, the first frame is set as the reference with an identity pose, then for each incoming frame *t*, matches are established with the previously rendered view t-1 and back-projected into the global coordinate system using the last estimated pose and the rendered depth map. This yields a set of 3D–2D correspondences used for Perspective-n-Point (PnP) registration to estimate the absolute pose. An error mitigation mechanism is implemented. When no keypoints are detected or RANSAC fails to validate the matched correspondences, the pose of the current frame cannot be estimated, and the current frame is skipped for expansion and mapping. In such cases, the system keeps optimizing only the previous views to refine the quality of the reconstruction, and tries to estimate the pose of the next frame from the last valid view.

**Fig. 3:**
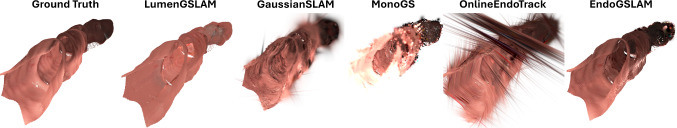
Online reconstruction methods tested on sigmoid_t2_a sequence of C3VD

### Online mapping

After estimating the pose of the new frame and adding new points, only the Gaussian and light parameters of the global map are optimized via gradient descent using a fixed window of keyframes, sampled based on their spatial ($\textit{s}_d$) and temporal ($\textit{s}_t$) distance from the current view, and minimizing a reconstruction loss.

Inspired by EndoGSLAM [11], the window is set to 25 views, and one frame every eight of the input stream is selected as a keyframe, with its ground-truth image, depth, and estimated pose retained. A probability function is then computed for all N keyframes, favoring those closer in space or time to the pose of the current view:

5 $$\begin{aligned} p_i = \dfrac{\log _2\Big ( 1 + \dfrac{s_d}{d_i + s_d/5} \Big ) + \log _2\Big ( 1 + \dfrac{s_t}{t_i + s_t/5} \Big )}{\sum _{j=1}^{N} \Big [ \log _2\Big ( 1 + \dfrac{s_d}{d_j + s_d/5} \Big ) + \log _2\Big ( 1 + \dfrac{s_t}{t_j + s_t/5} \Big ) \Big ]} \, (1 - p_c) \end{aligned}$$

where ${p_c}$, set to 0.1 as in [11], represents the probability associated with the current view. This approach prioritizes recent or nearby views for local optimization, while still sampling distant keyframes to maintain a globally consistent and up-to-date map.

### Gaussians and light optimization

The final loss combines photometric, depth, and tissue-consistency terms to improve image fidelity, enforce structural coherence, and produce physically plausible reconstructions. The photometric term balances pixel-level accuracy and perceptual similarity, with an L1 difference between rendered and ground-truth images contributing 80% and a structural dissimilarity (D-SSIM) term the remaining 20%. The depth term penalizes discrepancies between rendered and ground-truth depth maps using a robust Huber formulation. The tissue-consistency term, adapted from [10], encourages uniformity of optical parameters across structurally similar points by minimizing deviations in reflectivity, roughness, and albedo relative to their mean over all observed points.

The overall loss is expressed as a weighted sum of these components:

6 $$\begin{aligned} \mathcal {L} = \lambda _\text {photo} \, \mathcal {L}_\text {photo} + \lambda _\text {depth} \, \mathcal {L}_\text {depth} + \lambda _\text {tissue} \, \mathcal {L}_\text {tissue}, \end{aligned}$$

where $\lambda _\text {photo}$, $\lambda _\text {depth}$, and $\lambda _\text {tissue}$ balance the contributions of each term. Empirically, we observed that setting all weights to 1 provided a reasonable balance among the objectives; hence, all coefficients were fixed to 1 without further tuning. More details on each term can be found in Suppl. (Sec. A).

### Gradient scaler

To further preserve geometric and anatomical consistency, a per-parameter scheduler reduces the update magnitude for points that have already undergone multiple optimizations. A sigmoidal decay scheme, dependent on the number of times each Gaussian has been optimized, is applied independently to each point. As a result, frequently observed Gaussians receive progressively smaller updates, preventing overfitting to the current view and preserving structural fidelity.

## Experiments and results

### Datasets

The evaluation is conducted on the C3VD [18] and SCARED [19] datasets. For C3VD, we adopt the EndoGSLAM [11] preprocessing with an input resolution of $675 {\times } 540$. For SCARED [19], sparse depth maps are densified using surfel meshing, frames are resized to $640{\times } 512$, and points outside the available depth maps are actively pruned during optimization, as missing ground-truth depth can lead to divergence.

To further evaluate the robustness of our tracking method, we tested online models on a subsampled version of C3VD, retaining only one frame every five, thereby increasing inter-frame motion and mimicking realistic camera speeds.

### Experimental setup

To evaluate our model, we follow standard practice and withhold one every five frames in each sequence for testing. Performance of LumenGSLAM is compared with state-of-the-art offline and online methods based on Gaussian reconstruction. Specifically, Gaussian Pancakes [9], PR-ENDO [10], and 3DGS [8] are used as offline baselines, while EndoGSLAM [11], OnlineEndoTrack [20], GaussianSLAM [14], and MonoGS [13] serve as online benchmarks. All models are evaluated under the assumption that high-quality depth input is available. To ensure a fair and controlled comparison, ground-truth depth maps are provided to all frameworks, thereby isolating performance from depth estimation errors. For offline methods, our reconstruction serves as initialization. MonoGS is evaluated in both online and offline settings, while OnlineEndoTrack does not perform tracking, and ground-truth poses are used. To further benchmark tracking performance, results from ORB-SLAM3 [21] are also reported.

Reconstruction and Texture quality are evaluated using Peak Signal-to-Noise Ratio (PSNR), Structural Similarity Index (SSIM), Learned Perceptual Image Patch Similarity (LPIPS), and L1 distance between rendered and ground-truth depth maps (Depth-L1). Tracking performance is measured via Euclidean Absolute Trajectory Error (ATE) and Absolute Rotational Error (ARE), computed without alignment for Gaussian-based methods, as scale is assumed correct from the input depth maps, and with just scale correction for ORB-SLAM3.

LumenGSLAM was trained on a NVIDIA Tesla V100 GPU with 25 mapping-loop iterations, Adam optimizer, and a pruning interval of 50, following the settings of EndoGSLAM. Learning rates for Gaussian parameters followed the original 3DGS formulation, while those for the PBR model were set as in PR-ENDO. The scheduler follows a sigmoidal decay scheme with experimentally set parameters. For position and opacity, the slope is α=0.02 with a flex point at x=200; for covariance, scale, and base color, α=0.015 with an offset of x=500, enabling faster geometry freezing and smoother color optimization. Light parameters are optimized globally across the scene without a dedicated scheduler. Our model is tested with the proposed keypoint-based tracking (PnP) and with the same photometric approach of EndoGSLAM [11] (Ph).

**Table 1:** Reconstruction performance on C3VD and SCARED

–	Model	C3VD				SCARED			
		PSNR ↑	LPIPS ↓	SSIM ↑	Depth-L1 ↓	PSNR ↑	LPIPS ↓	SSIM ↑	Depth-L1 ↓
Offline	GaussianPancakes	30.09±4.28	0.31±0.07	0.84±0.08	–	22.64±1.51	0.25±0.07	0.80±0.07	–
	MonoGS (offline)	28.59±2.16	0.35±0.06	0.83±0.05	-	22.17±2.27	0.27±0.09	0.73±0.12	–
	PR-ENDO	**34**.**61**±**1**.**99**	0.24±0.05	**0**.**92**±**0**.**01**	–	**31**.**44**±**1**.**88**	**0**.**09**±**0**.**04**	**0**.**94**±**0**.**02**	–
	3DGS	32.03±4.03	0.28±0.05	0.90±0.03	–	21.44±3.49	0.39±0.08	0.71±0.10	–
Online	EndoGSLAM	25.26±3.69	0.30±0.05	0.85±0.05	$\underline{\boldsymbol{0.24\pm 0.07}}$	20.66±2.15	0.27±0.09	0.75±0.09	7.04±4.89
	GaussianSLAM	28.10±2.43	0.42±0.03	0.77±0.07	0.30±0.08	24.23±2.47	0.28±0.09	0.73±0.09	$\underline{\boldsymbol{0.52\pm 0.45}}$
	MonoGS (online)	19.25±6.17	0.46±0.09	0.72±0.15	1.93±1.94	17.39±2.04	0.32±0.09	0.62±0.11	5.81±3.03
	OnlineEndoTrack	19.13±3.51	0.49±0.03	0.67±0.07	1.26±0.61	17.93±1.82	0.27±0.09	0.67±0.12	6.62±5.23
	LumenGSLAM (Ph)	29.73±3.28	0.26±0.04	0.86±0.04	0.29±0.09	20.31±5.82	0.28±0.10	0.72±0.12	9.39±14.6
	LumenGSLAM (PnP)	$\underline{30.63\pm 3.39}$	$\underline{\boldsymbol{0.23\pm 0.03}}$	$\underline{0.89\pm 0.03}$	0.51±0.15	$\underline{27.57\pm 4.08}$	$\underline{0.14\pm 0.06}$	$\underline{0.90\pm 0.05}$	1.19±0.62

Underline represents best results among online methods, while bold stands for the absolute best

**Fig. 4:**
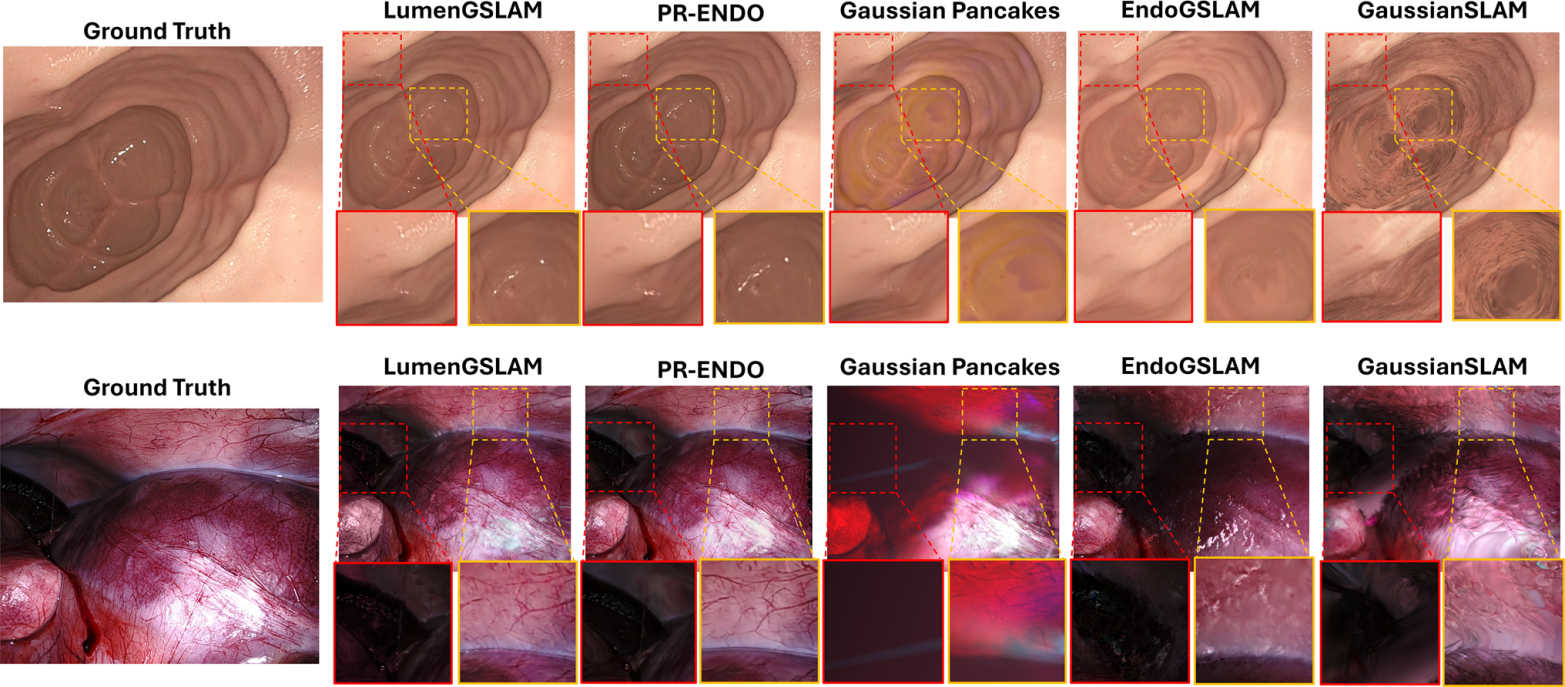
Qualitative rendering results on C3VD (top) and SCARED (bottom)

### Rendering results

Table 1 summarizes the performance of LumenGSLAM, compared with selected offline and online baselines. In terms of rendering, LumenGSLAM consistently outperforms all tested online models, including EndoGSLAM [11], which was designed for endoscopy (+5.4 PSNR, +0.04 SSIM, −0.07 LPIPS on C3VD; +6.9 PSNR, −0.13 LPIPS, +0.15 SSIM on SCARED).

Regarding Depth-L1, most models exhibit good robustness on C3VD, and only the proposed model and GaussianSLAM remain stable on SCARED. On C3VD [18], LumenGSLAM achieves the best overall LPIPS (0.23), thanks to its accurate online PBR modeling.

On C3VD, LumenGSLAM (PnP) reports a Depth-L1 of 0.51 mm, compared to 0.24 mm for EndoGSLAM and 0.30 mm for GaussianSLAM. This behavior is consistent with our optimization design. The introduction of per-Gaussian PBR modeling provides additional degrees of freedom, allowing photometric consistency to be achieved through adjustments in light and reflectance parameters, thereby reducing the need for aggressive geometric fitting. In parallel, the per-parameter gradient scheduler progressively limits updates for frequently observed Gaussians, stabilizing the map and preventing view-specific overfitting. As a result, the framework prioritizes structural stability and perceptual fidelity over sub-millimetric depth minimization.

Importantly, this effect does not indicate a systematic geometric limitation. On the more challenging SCARED dataset, LumenGSLAM (PnP) achieves a Depth-L1 of 1.19 mm, the second-best result among online methods and substantially lower than EndoGSLAM (7.04 mm), MonoGS (5.81 mm), and OnlineEndoTrack (6.62 mm). The slightly higher Depth-L1 on C3VD is therefore dataset-specific and likely related to the interaction between PBR-based optimization and the illumination characteristics of that dataset, rather than a fundamental weakness of the approach.

For other metrics, LumenGSLAM remains competitive (30.63 PSNR on C3VD and 0.14 LPIPS on SCARED). The best rendering results are obtained by PR-ENDO [10], which implements a similar light modeling but operates offline, leveraging the full video stream. In Fig. 4, the C3VD example illustrates how distant regions under reduced illumination are faithfully rendered only by LumenGSLAM and PR-ENDO, whose explicit light modeling accurately captures shadows and specularities. Similarly, in SCARED, only these models correctly capture both dark distant areas and fine vascular structures. Figure 3 shows full-sequence reconstructions. Thanks to surface-aligned initialization and per-parameter scaling, LumenGSLAM produces clean, artifact-free results, with uniform tissue appearance due to the decoupling of lighting and base color. See Suppl. Figure B2 and Suppl. video for additional qualitative results.

**Table 2:** Tracking performance on C3VD, a C3VD subset obtained by retaining one frame every five from the original sequences, and SCARED

Model	C3VD		C3VD (every 5)		SCARED	
	ATE [mm]	ARE [deg]	ATE [mm]	ARE [deg]	ATE [mm]	ARE [deg]
ORB-SLAM3	0.93±1.2	1.93±1.3	0.96±0.56	1.23±0.92	1.46±0.66	1.95±0.75
EndoGSLAM	0.89±0.46	1.19±0.58	9.32±11.92	35.65±23.31	41.84±69.52	24.23±42.30
GaussianSLAM	2.11±1.26	2.43±1.97	1.08±0.55	1.39±0.82	**1**.**15**±**1**.**19**	**0**.**76**±**0**.**88**
MonoGS (online)	3.86±2.85	4.35±3.56	9.90±6.61	12.29±9.92	15.07±8.05	24.48±25.78
LumenGSLAM (Ph)	**0**.**89**±**0**.**41**	0.99±0.34	9.74±9.92	18.40±20.81	9.04±12.69	16.37±26.43
LumenGSLAM (PnP)	0.93±0.59	**0**.**98**±**0**.**42**	**0**.**93**±**0**.**42**	**1**.**00**±**0**.**25**	2.02±1.46	2.14±0.75

**Fig. 5:**
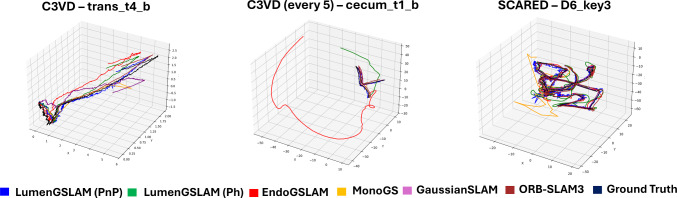
Qualitative trajectories on the 3 datasets

### Tracking results

Quantitative tracking performance is reported in Table 2, qualitative trajectories are shown in Fig. 5, while more trajectories are available in Fig. B1 of Suppl. and on Suppl. video. While ORB-SLAM3 [21] remains stable across all datasets, photometric methods prove to be competitive under slow and easy camera movements of C3VD with LumenGSLAM(Ph) and EndoGSLAM both reaching 0.89mm ATE. But, when under large inter-frame motion of C3VD (every 5) and complex trajectories of SCARED, they tend to diverge. Instead, when tested with the proposed Gaussian-coupled tracking, LumenGSLAM demonstrates superior robustness, reducing ATE and ARE by 8.81mm and 17.4$^{\circ }$ on C3VD (every 5) and by 7.02mm and 14.23$^{\circ }$ on SCARED. Notably, also GaussianSLAM [14], though purely photometric, remains robust to these challenges, likely due to the longer time spent mapping the current view. This helps photometric convergence, but, as shown in Figs. 3 and 4, it hinders the final reconstruction quality.

**Table 3:** The left block (**Mapping**) reports reconstruction ablation on C3VD: “n-init” (Gaussian initialization with surface normals), “n-loss” (normal-based loss regularization), “pbr” (explicit light modeling), and “sched” (per-parameter scheduler)

Mapping							Tracking		
n-init	n-loss	pbr	sched	**PSNR** ↑	**LPIPS** ↓	**SSIM** ↑	Model	**ATE** [mm]	**ARE** [deg]
				29.290	0.261	0.888	Constant + ph	9.742	18.397
✓				29.163	0.264	0.886	Identity + ph	8.641	16.701
	✓			29.189	0.265	0.885	PnP + ph	6.817	12.395
		✓		30.944	0.258	0.893	**PnP**	**0**.**932**	1.004
	✓	✓		27.831	0.318	0.799			
		✓	✓	30.565	0.226	0.887			
✓		✓		30.699	0.258	0.893			
✓	✓	✓	✓	29.112	0.238	0.884			
✓		✓	✓	**30**.**994**	**0**.**226**	0.893			

The right block (**Tracking**) evaluates tracking on C3VD using 1 frame every 5: “Constant” (constant speed initialization), “Identity” (next pose = previous one), “ph” (photometric refinement), and “PnP” (proposed tracking strategy)

### Ablation study

Table 3 summarizes the impact of key model components. Mapping results on C3VD show that the scheduler primarily enhances structural fidelity, reflected in higher SSIM, while PBR modeling and surface-aligned initialization improve visual quality, as indicated by increases in PSNR and reductions in LPIPS. Interestingly, using normals derived from depth gradients as regularization for the loss degrades performance when coupled to PBR, likely due to their approximate nature, which destabilizes the smoothness of normals obtained from rendering. Hence, normal regularization is not implemented. Analyzing tracking performance on C3VD (every 5 frames) clearly shows that photometric pose refinement is unstable under large inter-frame motion. Even when initialized with the proposed keypoint-based strategy, performance degrades significantly.

We further extend the ablation analysis of the tracking module to evaluate the impact of bundle adjustment (BA) across all considered datasets, including C3VD, its subsampled version (every 5 frames), and SCARED, with results reported in Table 4. Consistent across all datasets, incorporating BA to refine keyframe poses during mapping leads to only marginal variations in tracking accuracy.

This behavior can be attributed to the tight coupling between tracking and mapping in LumenGSLAM. Pose estimation at each frame relies on keypoint matching against the previously rendered view, which is itself generated from a Gaussian map continuously optimized over a sliding window of up to 25 keyframes. This multi-view local optimization enforces geometric consistency across overlapping views, yielding rendered references that are already locally coherent. Consequently, the 3D–2D correspondences used for PnP are grounded in a refined scene representation, inherently limiting drift accumulation over time. Under these conditions, the additional pose correction introduced by explicit BA provides limited incremental benefit relative to its computational cost. For this reason, bundle adjustment is not included in the final model.

**Table 4:** Impact of bundle adjustment (BA) on tracking performance across datasets measured in terms of ATE and ARE. ATE values are in millimeters, while ARE in degrees

Tracking	C3VD		C3VD (every 5)		SCARED	
	ATE [mm]	ARE [deg]	ATE [mm]	ARE [deg]	ATE [mm]	ARE [deg]
PnP + BA	$\mathbf {0.93\pm 0.54}$	$\mathbf {0.94\pm 0.33}$	0.95±0.58	$\mathbf {0.98\pm 0.30}$	$\mathbf {2.00\pm 0.99}$	2.35±1.15
PnP	0.93±0.59	0.98±0.42	$\mathbf {0.93\pm 0.42}$	1.00±0.25	2.02±1.46	$\mathbf {2.14\pm 0.75}$

### Reliance on depth-maps quality

To quantify the dependence of the proposed framework on the quality and scale of the input depth maps, Table 5 reports an evaluation of both the photometric (Ph) and the proposed keypoint-based (PnP) tracking strategies under controlled depth-scale variations and perturbations using the C3VD dataset. In particular, we analyze the behavior of the system under a fixed but non-metric depth scale, arbitrarily rescaled to emulate the scale ambiguity typical of monocular vision systems [21], as well as under two additional perturbation scenarios designed to simulate imperfect depth estimation.

The original C3VD frames are acquired with a maximum observable depth of 100 mm. To assess robustness to scale inconsistency, we rescale the maximum observable depth to 10 (while preserving relative depth relationships), thereby simulating the availability of depth maps with coherent structure but incorrect global scale, as commonly encountered in monocular settings. In this experiment, both the input depth maps and the corresponding ground-truth poses are uniformly rescaled by the same factor. As a result, pose estimation operates entirely within the rescaled coordinate system, and ATE is computed against ground-truth poses expressed in that same space.

**Table 5:** Evaluation on the reliance on input depth maps quality on C3VD. Depth-L1 and ATE values are reported in mm, whereas ARE values are in degrees

Track	Scale	Noise	Bias	Blur	PSNR ↑	Depth-L1 [mm] ↓	ATE [mm] ↓	ARE [deg] ↓
Ph	10 (fixed)	–	–	–	29.40	1.57	3.41	6.45
PnP	10 (fixed)	–	–	–	31.40	0.53	0.89	0.99
Ph	10 (fixed)	0.5%	0.1%	2px	29.50	1.15	3.21	7.64
PnP	10 (fixed)	0.5%	0.1%	2px	29.85	0.73	0.92	1.02
Ph	10 (fixed)	5.0%	1.0%	5px	23.90	7.44	150.9	59.70
PnP	10 (fixed)	5.0%	1.0%	5px	26.33	4.35	12.9	27.8
Ph	100 (real)	–	–	–	29.73	0.21	0.89	0.99
PnP	100 (real)	–	–	–	30.63	0.51	0.93	0.98

100 (real) refers to the actual maximum depth value in C3VD images, which corresponds to 100 mm, while 10 (fixed) is the arbitrary scale selected to degrade the depth maps’ absolute scale in this ablation study

In the “low” scenario, Gaussian noise and random bias are applied with standard deviations of 0.5% and 0.1% of the input scale, and Gaussian blur is set with a 2-pixel radius. In the “high” scenario, Gaussian noise and random bias have standard deviations of 5.0% and 1.0% of the input scale, with Gaussian blur set to a 5-pixel radius. While the system proves to be robust in terms of texture, these results clearly show that the proposed keypoint-based tracking strategy is better able to handle small perturbations, maintaining a 0.92 mm ATE under the “low” setting. Under the “high” scenario, both strategies diverge; however, thanks to the error mitigation mechanism for recovering failed pose estimates, the keypoint-based strategy maintains significantly greater robustness, with an ATE of 12.9 mm compared to 150.9 mm for photometric tracking. Interestingly, photometric tracking also appears sensitive to scale, as performance deteriorates when the scale is set to 10. This further underscores the susceptibility of photometric tracking and suggests that careful tuning of losses and learning rates is required when adapting to different scale scenarios.

### Limitations

LumenGSLAM enables robust and highly texturized reconstruction of explored cavities. However, its performance remains dependent on the reliability of local feature matching. In particular, SuperPoint may fail in cases of extreme tissue homogeneity or image degradation (e.g., blur or noise in the input depth maps), leading to pose estimation errors which, if not fully corrected by the error mitigation scheme, can degrade the final reconstruction quality. This suggests that domain-specific adaptation or fine-tuning of feature extractors could further improve robustness and generalizability.

In line with many recent state-of-the-art methods, LumenGSLAM adopts a non-end-to-end formulation and relies on dense depth information, typically obtained either through dedicated sensors or additional depth estimation modules. While this design facilitates stable geometry estimation and high-quality rendering, such conditions may not always be satisfied in standard clinical endoscopy, where depth is typically not directly available and must instead be inferred from monocular observations.

Although LumenGSLAM operates fully online, its optimization time is approximately $\sim 2\times$ longer than EndoGSLAM [11], primarily due to the additional computational overhead introduced by physically based rendering (PBR). Further acceleration may therefore require tighter system integration, for example by incorporating PBR directly into the CUDA-based rasterization pipeline.

## Conclusions

LumenGSLAM presents a novel RGB-D Gaussian-based online visual SLAM framework that integrates PBR into a unified, geometry-aware representation for endoscopic reconstruction and tracking. Through explicit light modeling and keypoint-based localization, it achieves anatomically consistent and photometrically accurate mapping, surpassing all online baselines and gaining +6.9 PSNR, −0.13 LPIPS and +0.15 SSIM over EndoGSLAM on SCARED. Despite rendering at over 100 fps, optimization stages, particularly per-Gaussian PBR computation, introduce additional overhead that hinders real-time performance. Therefore, future work will focus on runtime optimization, integration of a depth estimation block into the pipeline, and online tissue deformation modeling.

## Supplementary Information

Below is the link to the electronic supplementary material.

Supplementary file 1 (pdf 24577 KB)

Supplementary file 2 (mp4 39774 KB)

## Data Availability

Code is available in our GitHub.
