# Supplementary material for: LumenGSLAM: online physically based rendering with Gaussian Splatting for robust endoscopic reconstruction and tracking
Source: Int J Comput Assist Radiol Surg. 2026 May 13;21(6):1243–52. doi: 10.1007/s11548-026-03660-w (PMC13451309; doi:10.1007/s11548-026-03660-w)
Supplement: Supplementary file 1 [file 11548_2026_3660_MOESM1_ESM.pdf]

LumenGSLAM: Online Physically Based Rendering with Gaussian Splatting for Robust Endoscopic Reconstruction and Tracking

Francesco Leni[1,2*†], Chiara Lena[1*†], Zhehua Mao[2†], Sierra Bonilla[2], Luca Carlini[1], Danail Stoyanov[2], Elena De Momi[1], Sophia Bano[2]

[1*]Department of Electronics, Information, and Bioengineering, Politecnico di Milano, Via Giuseppe Ponzio, 34, Milan, 20133, Italy.
[2] UCL Hawkes Institute and Department of Computer Science, University College London, United Kingdom .

*Corresponding author(s). E-mail(s): francesco.leni@mail.polimi.it; chiara.lena@polimi.it;
†These authors contributed equally to this work.

Supplementary Materials

Appendix A Optimization

The final loss function used for LumenGSLAM optimization is a weighted sum of photometric, depth and tissue-consistency terms:

$$\mathcal{L} = \lambda_{\text{photo}}\,\mathcal{L}_{\text{photo}} + \lambda_{\text{depth}}\,\mathcal{L}_{\text{depth}} + \lambda_{\text{tissue}}\,\mathcal{L}_{\text{tissue}}, \tag{A1}$$

where λ_{photo}, λ_{depth}, and λ_{tissue} are scalar weights controlling the relative contribution of each term.

The photometric loss enforces consistency between the rendered image $\hat{C}(u)$, obtained from the Gaussian map, and the corresponding ground-truth RGB frame $C(u)$ from the selected keyframe. It combines a pixel-wise L1 term, promoting accurate color alignment, with a Structural Similarity (SSIM) term that captures perceptual

similarity by comparing local luminance, contrast, and structural patterns:

$$\mathcal{L}_{\text{photo}} = \sum_{u} \Big[0.8\,|\hat{C}(u) - C(u)| + 0.2\left(1 - \text{SSIM}(\hat{C}(u), C(u))\right)\Big], \tag{A2}$$

where λ_{ssim} controls the relative contribution of the SSIM component.

The depth loss compares the rendered depth map $\hat{D}(u)$ with the ground-truth depth $D(u)$. A pixel-wise Huber loss is adopted, behaving quadratically for small residuals and linearly for large ones, thereby increasing robustness to outliers and sensor noise:

$$\mathcal{L}_{\text{depth}}(\hat{D}(u) - D(u)) = \begin{cases} \frac{1}{2}(\hat{D}(u) - D(u))^2, & \text{if } |\hat{D}(u) - D(u)| \leq 0.2, \\ 0.2\,|\hat{D}(u) - D(u)| - \frac{1}{2}(0.2)^2, & \text{otherwise.} \end{cases} \tag{A3}$$

The tissue-consistency loss, adapted from [1], promotes local similarity among neighboring Gaussians in terms of albedo a_i, roughness r_i, and base reflectivity $F_{0,i}$, by penalizing deviations from their mean values across all N active Gaussians:

$$\mathcal{L}_{\text{tissue}} = \sum_{i=1}^{N}(a_i - \bar{a})^2 + \sum_{i=1}^{N}(r_i - \bar{r})^2 + \sum_{i=1}^{N}(F_{0,i} - \bar{F}_0)^2. \tag{A4}$$

Appendix B Timing analysis

Table B1 reports the runtime analysis of all the considered online approaches. The reported values account for the entire processing pipeline of each method, including tracking, mapping, optimization, and rendering stages whenever applicable.

For the proposed LumenGSLAM, we provide results both with and without the PBR module, which represents the most computationally demanding component of the pipeline. This comparison allows us to explicitly quantify the overhead introduced by physically-based rendering. We expect that a more efficient PBR implementation, achieved through direct integration into the CUDA-based rasterizer, could significantly reduce the computation time and further improve runtime performance.

Additionally, it is worth noting that all evaluated methods achieve a rendering speed higher than 100 FPS, ensuring real-time visualization performance across all configurations.

Table B1: *Runtime performance comparison across online methods.*

Method	Runtime Performance (s/frame)
OnlineEndoTrack	2.00
MonoGS (online)	0.91
GaussianSLAM	1.70
EndoGSLAM	0.50
LumenGSLAM (without PBR)	0.70
LumenGSLAM (with PBR)	1.40

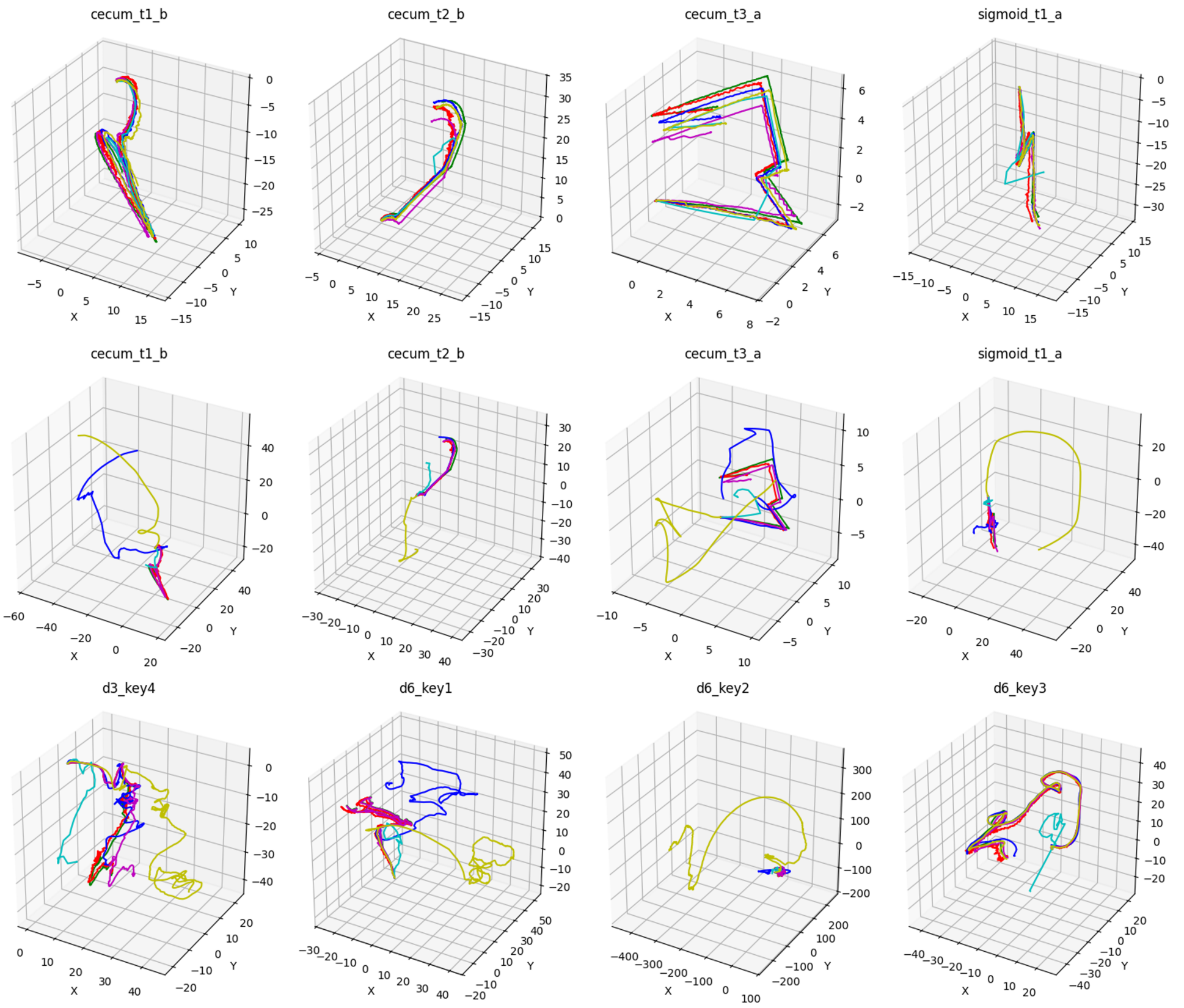

Fig. B1: *Qualitative tracking results on C3VD (first row), C3VD (every 5) second row and SCARED (last row). GT trajectory is shown in green, LumenGSLAM (PnP) in red, LumenGSLAM (Ph) in blue, GaussianSLAM in purple, MonoGS in cyan, and EndoGSLAM in yellow.*

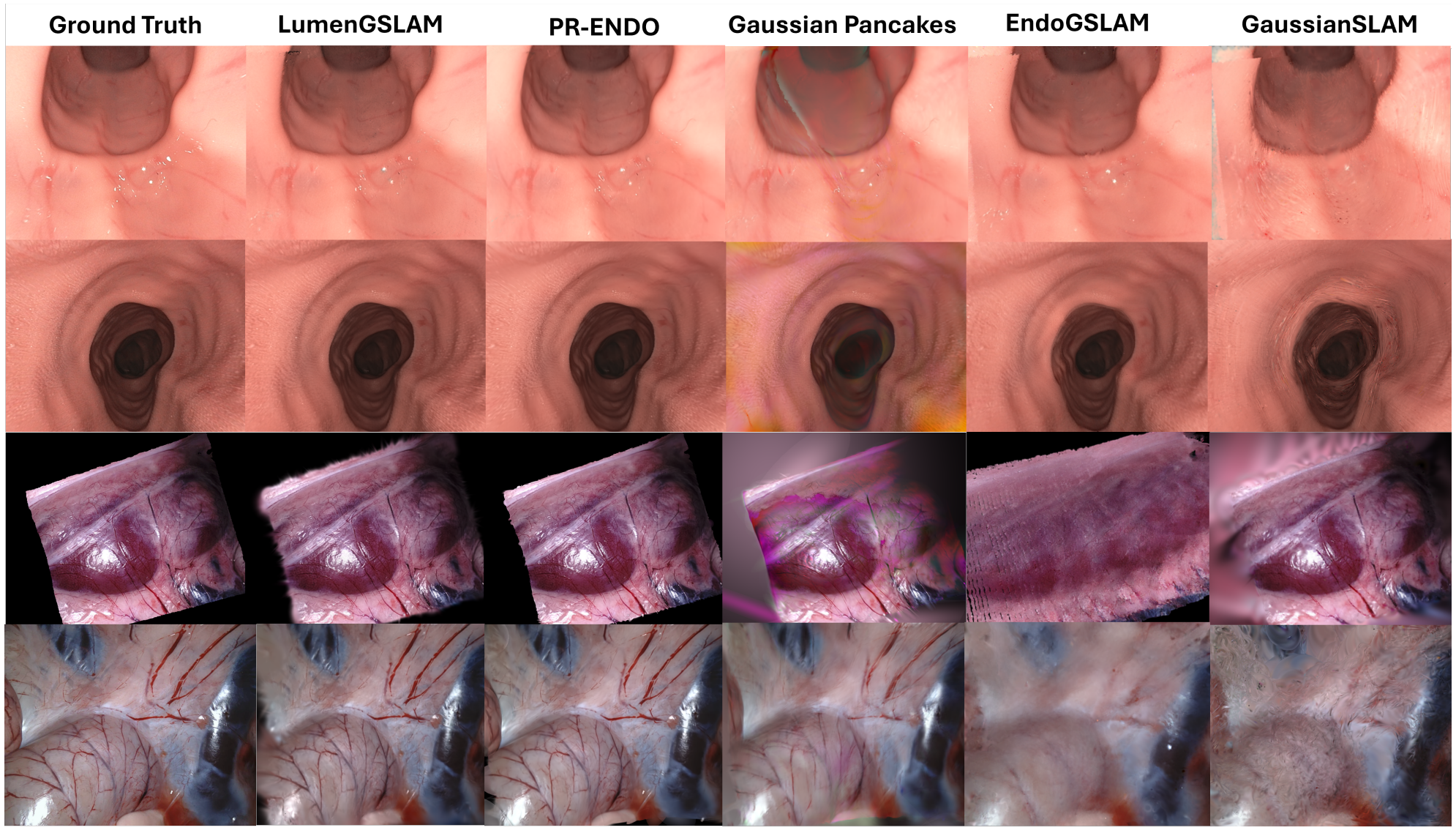

Fig. B2: *Qualitative Rendering results on* `trans_t2_c` *and* `sigmoid_t2_a` *sequences of C3VD on the first 2 rows and* `d1_key1` *and* `d6_key3` *of SCARED in the last two rows.*

References

[1] Kaleta, J., Smolak-Dyżewska, W., Malarz, D., Dall'Alba, D., Korzeniowski, P., Spurek, P.: Pr-endo: Physically based relightable gaussian splatting for endoscopy. In: International Conference on Medical Image Computing and Computer-Assisted Intervention, pp. 391–401 (2025). Springer
